# Supplementary material for: How do we engage people in testing for COVID-19? A rapid qualitative evaluation of a testing programme in schools, GP surgeries and a university
Source: BMC Public Health. 2022 Feb 14;22:305. doi: 10.1186/s12889-022-12657-4 (PMC8842975; doi:10.1186/s12889-022-12657-4)
Supplement: Supplementary file 1 — Additional file 1. [file 12889_2022_12657_MOESM1_ESM.zip › Saliva testing discussion guide declinerR4.docx]

**Southampton Covid-19 testing programme - Decliner**

Hello, I’m [Name] from the Southampton Covid-19 testing programme. I understand that you were invited to take part in the Southampton Covid-19 testing programme, but felt unable to. You have, however, kindly consented to taking part in a telephone interview about your reasons for not taking part. I am phoning you today to either speak to you now or to arrange another time, if that is still ok with you?

If yes, move onto interview guide

If no, when is a good time to talk? (arrange date and time and confirm phone number)

Thank you for agreeing to take part in this phone interview. We are also going to be audio-recording the conversation, with their permission. After the interview, our team will type up everything that you have said, and when we have spoken to everyone, we will produce a summary of all the interviews for a written report about reasons for not taking part in the Southampton Covid-19 testing programme. The recordings will be deleted after we produce the report. The transcripts and reports will not contain any names, or anything that will identify you.

Once you have done this interview, we will send you a £20 amazon voucher to say thank you for your time.

I will begin by asking you to confirm your name, but your name and your household member names will not be reported at any point. So please be assured that your contribution today will remain confidential in the reports.

The interview will last approximately 30 minutes. If you wish to leave the conversation at any point, you are of course able to do that.

Thank you …

If it is okay with you, I would like to record this interview. [START RECORDING]

***Verbal assent:*** Are you happy to take part in this study and for it to be recorded?

**Prompting questions for each participant:**

- What have you heard about the testing programme? What were your initial thoughts?
- How were you approached about taking part in the programme? *Prompt: the information, the approach*
- What were the main things that made you not take part in the testing programme?
- What could we have changed that might have made you take part?
- What do you think might be the main benefits to taking part in the testing programme?
- What would you say to a friend about taking part in the testing programme?
- Following this interview, how do you feel now about the testing programme?

**End with:**

- Is there anything else you would like to say about research or to ask me about?

Thank you for your time.

What’s your email address so we can send you the amazon voucher?
