# Supplementary material for: How do we engage people in testing for COVID-19? A rapid qualitative evaluation of a testing programme in schools, GP surgeries and a university
Source: BMC Public Health. 2022 Feb 14;22:305. doi: 10.1186/s12889-022-12657-4 (PMC8842975; doi:10.1186/s12889-022-12657-4)
Supplement: Supplementary file 1 — Additional file 1. [file 12889_2022_12657_MOESM1_ESM.zip › Saliva testing discussion guide implementorR4.docx]

**Southampton COVID-19 Saliva Testing Programme – Implementor**

Hi NAME,

I am XXXXX from Southampton Covid-19 testing programme. Thank you for agreeing to speak to me about your experiences of setting up and running the testing programme.

With your permission, I will be audio-recording the discussion. After the interview, our team will type up everything that you have said, and when we have spoken to everyone, we will produce a summary of all the interviews for a written report about what it is like to be involved in the Southampton Covid-19 testing programme. The recordings will be deleted afterwards. The transcripts and reports will not contain any names, addresses, or anything that will identify you.

I will begin by asking you who you are, but your name will not be reported at any point. So please be assured that your contribution today will remain confidential in the reports.

The interview will last approximately 30 minutes. If you wish to leave the discussion at any point, you are of course able to do that.

Thank you …

If it is okay with you, I would like to record this interview. [START RECORDING]

***Verbal assent:*** Are you happy to take part in this study and for it to be recorded?

**Interview guide**

1. When you were approached about the Southampton saliva testing programme, what did you first think?
2. What has been your involvement in the Southampton saliva testing programme?
3. What is your role in the testing programme?
4. What have you and your team had to do to set up the programme?
5. What tasks do you and your team do on a weekly basis to keep the programme running?
6. What procedures have you put in place if a participant get a positive test? Prompts: support
7. How do you engage with staff, students, and others across your organisation about the programme? What is the community perspectives like about the testing programme? What feedback have you had from your staff/parents/students?
8. What are the challenges with the testing programme?
9. How could the processes be improved?
