# Supplementary material for: How do we engage people in testing for COVID-19? A rapid qualitative evaluation of a testing programme in schools, GP surgeries and a university
Source: BMC Public Health. 2022 Feb 14;22:305. doi: 10.1186/s12889-022-12657-4 (PMC8842975; doi:10.1186/s12889-022-12657-4)
Supplement: Supplementary file 1 — Additional file 1. [file 12889_2022_12657_MOESM1_ESM.zip › Saliva testing discussion guide participantR4.docx]

**Southampton Saliva Testing Programme - University students**

Hello welcome to the focus group about your experience of the Southampton COVID-19 Testing Programme.

Thank you for agreeing to take part in this focus group discussion. I’m NAME and I’ll be facilitating the discussion today and my colleague NAME will be making notes and keeping an eye on the time.  We are also going to be audio-recording the discussion. After the discussion our team will type up everything that you have said, and when we have spoken to everyone, we will  produce a summary of all the discussions we have had with students for a written report about what it is like to be involved in the Southampton Saliva testing programme.  We would like to know what has gone well, what the challenges have been and also how to improve the programme. The recordings will be deleted afterwards. The transcripts and reports will not contain any names, addresses, or anything that will identify you.

Once you have done this focus group, we will send you a £20 amazon voucher to say thank you for your time. In order for you to receive your amazon voucher, please send OBSERVER NAME a private message on Zoom with your email address. If you have any issues we can solve them at the end.

I will begin by asking you for your names. Be assured this is for the purposes of the discussion only. Your names will not be reported at any point. So please be assured that your contribution today will remain anonymous and confidential in written reports.

We encourage you to have your cameras on but we understand if you would prefer to have them off. If you want to remain anonymous you can change your name at the bottom under participants. Mainly you will have your microphones on mute, but do unmute when you would like to talk. You can also use the chat function if you would like to speak and raise your hand physically or on Zoom. It would be great if we could hear from all of you.

The discussion will last approximately 1 hour. If you wish to leave the discussion at any point, you are of course able to do that.

Thank you …

If it is okay with you, I would like to record this interview. [START RECORDING]

***Verbal assent:*** Are you happy to take part in this study and for it to be recorded?

We will start by quickly going round and introducing ourselves. Just say your name, what year you are in, and if you live in halls, shared living or other.

**Interview guide (select 10 questions)**

1. What is your experience of the Southampton COVID-19 testing programme so far?
2. What had you heard about the testing programme before you registered?
3. What were your initial thoughts when you were asked to take part?
4. How was your registration onto this programme ? *Prompts: motivation/ pros and the cons*
5. What is your experience of the tests being delivered and returned? (if drop off point is inconvenient, ask which area they live in and their nearest drop off point)
6. How has it been taking the test? *Prompts: How often do you take it?*
7. How have you found labelling specimen pots and bags?
8. What is your experience of getting the results? *Prompts: negative and positive*
9. If your test result was to come back positive, how do you feel about household isolation? How likely are all household members to comply? Changed plans?
10. How has the communication been with the testing team?
11. What do the students you know generally think about the saliva testing programme?
12. What is stopping some students registering for the programme?
13. What would make these students more likely to register?
14. Any last comments or thoughts that we haven’t yet discussed?

**Ask the observer if they have any questions or comments**

**Thank you for taking part in this focus group, your insight has been invaluable.**
